# Fergus Shanahan: The language of illness: Liberties Press, 2020, (ISBN (print): 978-1-912589-15-9 and ISBN (e-book): 978-1-912589-16-6)

**DOI:** 10.1007/s11845-021-02568-5

**Published:** 2021-03-09

**Authors:** William P. Tormey

**Affiliations:** University of Ulster Coleraine, Coleraine, Northern Ireland UK

**Keywords:** Communication, Philosophical, Tradition

## Abstract

"The language of illness" is a wide ranging exploration of the therapeutic medical process - where it goes right and where is misfires. Communication and empathy are core values explored in the detail exemplifying the art of medicine along side the science. There is a deep seated wisdom and sympathetic philosophy within its pages which is very valuable for the healing and caring professions.

www.casemateipm.com

Dr Fergus Shanahan and I are of similar age and share Dublin City and University College Dublin as core environmental influences in our youth. Dublin was a city of world famous medical giants especially in the nineteenth century. Illuminati included Dominic Corrigan (1802–1880), William Stokes (1804–1878), Sir William Wilde (1815–1876), and Oliver St John Gogarty (1878–1957). The latter, referred to in James Joyce’s ‘Ulysses’ as Buck Mulligan, featured in the greatest novel in the English language in the twentieth century.

The opening words of Ulysses are ‘Stately, plump Buck Mulligan came from the stairhead, bearing a bowl of lather on which a mirror and a razor lay crossed. A yellow dressinggown, ungirdled, was sustained gently behind him on the mild morning air. He held the bowl aloft and intoned’:

*- Introibo ad altare Dei.*

Idealisation of a ‘doctor’ germinated in my mind and psyche in the 1960s driven by the novels of AJ Cronin (who was the progenitor of Dr Finlay’s Casebook on BBC television (monochrome) (1961–1971)) and Marcus Welby MD (1969 to 1976), a kindly family doctor in the US series played by Robert Young and shown on RTE television.

Empathy for the feelings and fears of patients were not high on the agenda at medical school in the era of the clinical apprentice. I vividly remember DK O’Donovan. Professor of Medicine recommending that the class read ‘Malone meurt’ by Samuel Beckett published in French in 1951 which addresses Malone’s journey to death. It was such an unusual recommendation and chimed with the 1960s era.

For medical, nursing and paramedical staff, I strongly recommend “The language of illness” because this book is a veritable ‘tour de force’ of the doctor/patient interactions in the medical treatment sphere, both positive and negative.

The subject matter of the chapters rings so true. Examples are ‘Disease speak and its distancing effect’; ‘The illness words’'; ‘Its the way you say it’; ‘No words just silent signals’; ‘Healing words, hurting words’; ‘Words that turn people into patients’; and ‘the weight of a word: caring, dignity and empathy’. There is an extensive bibliography which is useful for those in training and those on master’s degree courses in the caring professions.

To have a book that comprehensively observes doctoring with acute observations all the way through based on a long medical career in direct patient care in clinical medicine combined with a stellar original science career in gastroenterology is decidedly unusual. By birthright, this author joins the greats of Dublin medicine even if claimed by Cork.

Buy this book.

